# Severe Sporotrichosis Caused by *Sporothrix brasiliensis*: Antifungal Susceptibility and Clinical Outcomes

**DOI:** 10.3390/jof9010049

**Published:** 2022-12-28

**Authors:** Vivian Fichman, Fernando Almeida-Silva, Dayvison Francis Saraiva Freitas, Rosely Maria Zancopé-Oliveira, Maria Clara Gutierrez-Galhardo, Rodrigo Almeida-Paes

**Affiliations:** 1 Laboratory of Clinical Research in Infectious Dermatology, Evandro Chagas National Institute of Infectious Diseases (INI), Oswaldo Cruz Foundation (FIOCRUZ), Rio de Janeiro 21040-360, RJ, Brazil; 2 Laboratory of Mycology, Evandro Chagas National Institute of Infectious Diseases (INI), Oswaldo Cruz Foundation (FIOCRUZ), Rio de Janeiro 21040-360, RJ, Brazil

**Keywords:** sporotrichosis, amphotericin B, antifungal susceptibility, minimal inhibitory concentration, synergism, alcoholism

## Abstract

Itraconazole is the first choice for treating sporotrichosis. Amphotericin B is indicated for severe and disseminated forms. The aim of the study was to evaluate the antifungal susceptibility of *Sporothrix brasiliensis* strains isolated from patients with severe sporotrichosis treated with amphotericin B and correlate with clinical outcomes. Clinical and epidemiological data were obtained from severe sporotrichosis cases caused by *S. brasiliensis*. Antifungal susceptibility tests against amphotericin B, itraconazole, terbinafine, posaconazole, and 5-flucytosine were performed. Moreover, possible synergisms between amphotericin B and posaconazole or 5-flucytosine were assessed. Relationships between clinical and laboratorial data were then analyzed. Forty-six *S. brasiliensis* isolates from 37 patients were studied. Clinical forms included disseminated (94.6%) and disseminated cutaneous sporotrichosis (5.4%). The median treatment time was 784 days (range: 7 to 3115 days). Cure occurred in 45.9% of the cases and death due to sporotrichosis in 24.3%. Forty-three (93.5%) *S. brasiliensis* isolates were classified as wild-type for all the antifungals tested according to their in vitro antifungal susceptibility. There was no synergism for the combinations studied. Finally, we found no association between higher Minimal Inhibitory Concentration (MIC) values of amphotericin B or itraconazole with unfavorable outcomes; however, there were higher MIC values of itraconazole in strains isolated from alcoholic patients. Possibly, clinical factors, such as the extent of dissemination, immunosuppression, and late treatment onset, are the main determinants of patient outcomes, rather than antifungal resistance. The current study suggests that the need to use amphotericin B therapy is not associated with the emergence of *S. brasiliensis* resistant strains.

## 1. Introduction

Sporotrichosis is a subcutaneous mycosis caused by dimorphic fungi from the genus *Sporothrix*. The mycosis occurs worldwide; however, major endemic areas are in the tropical and subtropical zones. Sporotrichosis is classically acquired after traumatic inoculation with soil and plant debris harboring the fungus, or alternatively, through bites and scratches of a series of animals, especially domestic cats, which characterizes the zoonotic transmission, currently the main one in Brazil [1,2]. 

Itraconazole (ITR) is the first therapeutic choice for cutaneous sporotrichosis. Terbinafine (TRB) and potassium iodide are alternative options, the former with fewer drug interactions, and the latter with low cost. Treatment must be held until complete clinical cure [3,4]. Amphotericin B (AMB) is indicated for severe forms, especially extracutaneous or disseminated sporotrichosis. This drug must be administered until significant clinical improvement is achieved, followed by ITR, which must be maintained until a minimum period of one year, when treatment is completed [3]. Although AMB was introduced in the 1950s, it remains the only parenteral antifungal drug recommended for the treatment of severe sporotrichosis. 

AMB belongs to the family of polyene macrolide antibiotics, with fungicidal action. It is cardio and nephrotoxic, and infusion reactions can occur. There are three main presentations commercially available in most countries where sporotrichosis is endemic: deoxycholate, lipid complex, and liposomal. The last two presentations are better tolerated [5]. AMB dispersion was another lipid formulation; however, it is no longer commercially available since it was discontinued in 2011 due to high rates of infusion-related reaction [6]. One study presenting 73 patients with severe sporotrichosis treated with AMB showed that 45.2% relapsed after initial improvement, represented by the number of AMB cycles performed. In addition, there was a high rate of adverse events, leading to treatment interruption in 20.5% of the cases; the most serious events were caused by the deoxycholate formulation [7].

There are several studies describing *Sporothrix* spp. susceptibility to antifungal drugs [8,9,10]. Internationally, the broth microdilution, recommended by the Clinical & Laboratory Standards Institute (CLSI), is the main protocol used for the in vitro determination of Minimal Inhibitory Concentrations (MICs) for drugs used in sporotrichosis treatment. Epidemiological cutoff points were established for *Sporothrix schenckii* and *Sporothrix brasiliensis* using this method [8]. These cut-off points could help clinicians to choose the best treatment for each patient. However, there is a lack of studies associating the MIC of the drugs used in the sporotrichosis treatment with the outcomes of each patient. Thus, we evaluated the antifungal susceptibility of *S. brasiliensis* strains isolated from a cohort of patients with severe sporotrichosis treated with AMB and correlated with treatment length, overall survival, and other clinical variables.

## 2. Materials and Methods

This study was approved by the Research Ethics Committee of the Instituto Nacional de Infectologia Evandro Chagas (INI), Fundação Oswaldo Cruz (FIOCRUZ), Rio de Janeiro, Brazil (CAAE: 08625819.0.0000.5262). An observational, retrospective study was carried out with cases seen from 1998 to 2018 at the INI/FIOCRUZ, a referral center for sporotrichosis in the state of Rio de Janeiro, Brazil. Adult patients diagnosed with sporotrichosis by isolation of *S. brasiliensis* in clinical specimens, treated with AMB, associated with other drugs, and with at least one viable isolate stored in the mycology laboratory were included. The fungal species was determined through molecular identification methods performed using the specific primers targeting the calmodulin gene [11].

All the patients completed a standard clinical and laboratory assessment at admission, which was periodically repeated according to the individual clinical indications. A protocol for the investigation of systemic involvement is performed in the cases of suspected extracutaneous/widespread disease. This investigation includes fungal cultures from sputum, blood, urine, cerebrospinal fluid, or bronchoalveolar lavage (BAL). In addition, rhinolaryngoscopy, fundoscopy, and radiographies of the chest and bones (hands, feet, and long bones for the investigation of osteomyelitis) are also performed to evaluate the mycosis severity. Clinical and epidemiological data were obtained from the medical records. Data were anonymized/de-identified to protect the patient’s privacy and confidentiality.

MICs of AMB, ITR, posaconazole (POS), TRB, and 5-flucytosine (5-FC) against all *S. brasiliensis* isolates obtained from the included patients were determined using the M38-A2 protocol (CLSI, 2008); and analyzed according to the epidemiological cutoff values (ECV) precluded for *S. brasiliensis* and the antifungal drugs used for sporotrichosis treatment [8]. MIC values were determined at least in duplicate for each strain.

The assessment of possible synergisms between AMB and 5-FC or POS was performed using the checkerboard method to determine the fractional inhibitory concentration index (FICI). Briefly, 5-FC or POS (called drug A) were serially diluted (7 dilutions), from 8 to 0.12 µg/mL, in 96-well plates; and each dilution applied to the rows of the plate. AMB (called drug B) was serially diluted (10 dilutions), from 8 to 0.015 µg/mL; and each dilution applied to columns of the same 96-well plate. FICI was defined as (MIC of the drug in combination/MIC drug A alone) + (MIC of the drug in combination/MIC drug B alone). Synergism was categorized as follows: synergistic effect, FICI ≤ 0.5; antagonist effect, FICI > 4; no effect, FICI > 0.5–4 [12]. This experiment was performed in duplicate with 15 randomly selected strains among those included in the study.

A comparison was made between the survival curves of patients presenting isolates with different MIC values, using the Mantel-Cox test and the GraphPad Prism 8.4 program. *p*-values < 0.05 were considered significant. For ITR, the MIC cutoffs used to construct the survival curves were 0.5 µg/mL and ≥1 µg/mL. For AMB, the MIC cutoffs used to construct the survival curves were ≤1 µg/mL and ≥2 µg/mL. These cutoff points were chosen to include sufficient patients in each group to perform the statistical tests. The Mann–Whitney test was used to compare MIC values for AMB and ITR according to the following variables: sex, ethnicity, zoonotic transmission, HIV coinfection, diabetes, alcoholism, tuberculosis coinfection, and the AMB formulation used (deoxycholate or lipid complex). A multiple Cox survival model was performed with the same ITR and AMB MIC cutoff values used for survival curves and controlling by HIV coinfection. *p*-values < 0.05 were considered significant.

## 3. Results

### 3.1. Strains

Forty-six *S. brasiliensis* isolates from 37 patients with severe sporotrichosis were studied. The fungi were isolated from the skin (97.3%), mucosal membranes (27.0%), sputum (24.3%), cerebrospinal fluid (13.5%), blood (10.8%), synovial fluid (8.1%), BAL (8.1%), lymph node, bone marrow aspirate and urine (5.4% each), and from a bone biopsy (2.7%). Seven patients had isolates from more than one clinical specimen or from different dates.

### 3.2. Patients’ Data

Clinical and epidemiological data of the patients are described in Table 1. Most patients were male (81.1%), non-white (75.7%), and with up to seven years of schooling (68.8%). The mean age was 45.95 years. Regarding possible sources of infection, 25 patients (67.6%) reported contact with cats. Among them, 16 reported that the cat was sick. The median time between symptom onset and admission was 90 days (range: 30–2190 days). The main comorbidities presented were HIV coinfection (59.5%), alcoholism (37.8%), diabetes mellitus (21.6%), and tuberculosis coinfection (16.2%). Clinical forms included disseminated (94.6%) and disseminated cutaneous sporotrichosis (5.4%).

### 3.3. In Vivo Response to Treatment

All the patients used AMB to treat sporotrichosis at some point of their follow up in our institution. As presented in Table 1, the median cumulative dose of AMB was 1400 mg of deoxycholate (range: 50 to 11,250 mg) and 5150 mg of lipid complex formulation (range: 200 to 13,900 mg). Additionally, ITR, POS, and TRB were also used during the treatment in 36, 10, and 8 patients, respectively. The median treatment time was 784 days (range: 7 to 3115 days). Cure occurred in 45.9% of the cases and death due to sporotrichosis in 24.3%. Other outcomes were loss to follow up (8.1%) and death unrelated to sporotrichosis (5.4%). At the end of the study, 16.2% of the patients were still on treatment.

### 3.4. In Vitro Antifungal Susceptibility

Table 2 summarizes the MICs of the 46 *S. brasiliensis* isolates. Forty-three were classified as wild-type for all the antifungals tested according to their in vitro antifungal susceptibility. For these wild-type isolates, the MIC values ranged from 0.5 μg/mL to 2 μg/mL for AMB; 0.03 µg/mL to 0.12 µg/mL for TRB; 0.5 µg/mL to 2 µg/mL for ITR; 0.5 μg/mL to 2 μg/mL for POS, and ≥8 μg/mL for 5-FC, which does not have a defined ECV. There were two isolates non-wild-type for TRB, with MIC of 1 µg/mL and 0.25 µg/mL; however, they were wild-type for the other antifungals tested. One isolate was non-wild-type for all the antifungals tested, with MIC of 8 μg/mL for AMB, ITR, POS, and 5-FC; and 0.5 μg/mL for TRB. Synergism was studied in 15 *S. brasiliensis* strains, which correspond to 33% of the total isolates. We did not find synergism or antagonism between AMB and POS, or between AMB and 5-FC in the evaluated isolates (FICI = 2 for all the strains and drug combinations).

### 3.5. Relationships between Clinical and Laboratorial Data

**Figure 1:**
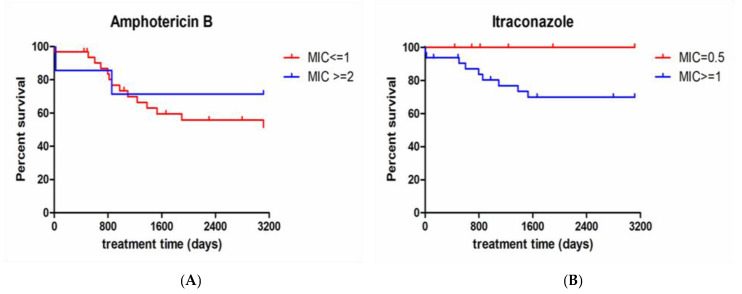
(**A**) Comparison between survival curves of patients presenting isolates with MIC ≤ 1 and MIC ≥ 2 for amphotericin B. *p* value = 0.455 (Mantel-Cox test). (**B**) Comparison between survival curves of patients presenting isolates with MIC = 0.5 and MIC ≥ 1 for itraconazole. *p* value = 0.194 (Mantel-Cox test); MIC (µg/mL).

The patient infected with the non-wild-type isolate had disseminated sporotrichosis, with cutaneous, bone, ocular, upper airways, and central nervous system involvement, having received high doses of AMB (400 mg of deoxycholate and 13,500 mg of lipid complex); and died due to sporotrichosis after 856 days of treatment. In addition, eight patients infected with wild-type isolates died due to sporotrichosis, indicating a lethality of 22.2% in patients infected with wild-type strains. In patients with more than one isolate studied, the MIC values varied at most by one dilution, except for the patient infected with a non-wild-type isolate, described before. This patient had one non-wild-type isolate from the cerebrospinal fluid, but also a wild-type isolate from sputum with an MIC of 1 μg/mL for AMB and ITR, 0.12 μg/mL for TRB, and 2 μg/mL for POS. Higher MIC values for ITR were more prevalent among the alcoholic patients (Table 3). We found no association between the MIC values above 1 μg/mL for AMB (*p* = 0.456) and above 0.5 μg/mL for ITR (*p* = 0.194) with the survival rates of the included patients (Figure 1). In the multiple Cox model, there was no association between lower ITR and AMB MIC values and higher cure risk (Table S1).

## 4. Discussion

In the current hyperendemic sporotrichosis in Rio de Janeiro, Brazil, which has lasted for more than 20 years, it has been reported that *S. brasiliensis* is the most virulent species that is related to cases of zoonotic transmission, and responsible for atypical and more severe clinical manifestations [13,14,15]. This study corroborates these findings, presenting 37 patients with severe forms of sporotrichosis infected by this species. They are part of a larger case series study that included 73 patients with severe sporotrichosis [7].

Among the isolates herein studied, only one was considered non-wild-type against all the tested drugs, which was expected, since the frequency of *Sporothrix* spp. non-wild-type isolates is low [8,9]. This patient presented a disseminated disease, received high doses of AMB, and died due to sporotrichosis complications. However, susceptibility to AMB and other drugs in wild-type strains did not prevent unfavorable outcomes, as lethality was high even in patients infected with wild-type strains. Since the two patients with isolates non-wild-type for TRB were treated with AMB and ITR, and did not use TRB, it was not possible to assess the impact of this finding on their treatment response.

While other systemic mycoses usually have a good response to AMB [16,17], severe sporotrichosis has many cases of unsatisfactory response [7], especially when the central nervous system is affected, which is a site of difficult sterilization in patients with sporotrichosis [18]. For this reason, we evaluated possible synergisms of AMB with two antifungal drugs. 5-FC was chosen because its combination with AMB is recognized as synergistic for other mycoses [19,20,21]. POS was chosen because it is the first oral antifungal option, in our institution, to be associated with AMB, for refractory cases of sporotrichosis meningitis due to the severity of this clinical form, since ITR absorption is erratic; and it does not have good penetration into the central nervous system. Approximately one third of the strains in the study were tested and we found no synergisms, pointing that these drugs, in combination, do not appear to have a broad spectrum of action against *S. brasiliensis*, and evidencing the difficulty to treat severe sporotrichosis cases.

Alcoholism was found to be associated with higher MIC values for itraconazole. Qualitative studies have explored the alcohol’s effect on antiretroviral treatment (ART) adherence. Participants reporting hazardous or harmful alcohol use were significantly more likely to either stop, miss, forget, or be careless about their ART than their counterparts without alcohol problems. By making it more difficult to adhere to ART, alcohol use could also interfere with viral load suppression and increase the risk of becoming resistant to ART [22,23]. Similar mechanisms could be involved here with itraconazole treatment. In addition, ethanol alters the expression or activity of some enzymes used in the biotransformation of various drugs. In chronic use, it can induce the biotransformation of other drugs metabolized by the cytochrome P450 enzyme system [24], leading to subtherapeutic levels of itraconazole and favoring the emergence of resistance.

Previous studies recommend a 1 µg/mL threshold to define the AMB resistance of different fungi [25]. However, we have not identified differences when correlating it with the outcomes of patients included in this study. Higher thresholds are often found for *Sporothrix* spp. [8,9,10]. Some potential correlations might be identified when using higher MIC thresholds to separate groups of patients with sporotrichosis; however, our study did not feature enough isolates to support statistical analyses with more extreme MIC values. It is a challenging task to obtain a sufficiently large group to enable statistical tests with sufficient power, due to the low frequency of severe sporotrichosis cases requiring treatment with AMB and the low occurrence of non-wild-type strains, usually under 5% [8].

As most patients were treated with ITR, resistance to this medication might have also been a determinant outcome factor. We use a 0.5 µg/mL MIC cutoff for the comparison of ITR values and outcomes. This cutoff enabled sufficiently large patient groups across both sides (MIC = 0.5 µg/mL and MIC ≥ 1 µg/mL). We failed, however, to find a significant survival difference among the groups. Clinical factors, such as the extent of infection dissemination, immunosuppression, and late treatment onset are probably the main determinants of patient outcomes [7]. In addition, it is important to state that the in vitro findings may be different from the in vivo experience. Itraconazole has erratic absorption and interacts with several drugs, leading to subtherapeutic levels.

One patient presented two isolates, one obtained eight months later than the first. This time did not interfere in the MIC values for ITR, AMB, TRB, POS, and 5-FC. Another study from our research group compared antifungal susceptibility between isolates from single patients. These isolates were obtained across intervals ranging from two months to five years. The study showed that long treatment periods are not associated to the emergence of azolic resistance [26]. The current study suggests that AMB therapy is not associated with the emergence of in vivo *S. brasiliensis* resistance during treatment. A larger study, with more patients, is necessary to support this finding.

Six patients had more than one isolate collected within a two-month window. Among them, MIC values varied by at most one dilution. The single patient infected by a non-wild-type strain also presented a wild-type isolate from another clinical sample, demonstrating the complexity of sporotrichosis in severe cases; and the need for susceptibility, virulence, and genetic studies of multiple isolates from the same patient, especially those obtained from unusual sites such as blood and cerebrospinal fluid.

In summary, we did not identify an association between higher MIC values and patient outcomes in severe sporotrichosis. This means that, as it occurs with other mycoses [27], antifungal susceptibility results is not the sole guide for a successful sporotrichosis therapy. This study presents limitations inherent to a retrospective study, with a limited sample size and carried out at a single reference center. Further studies of AMB treatment in sporotrichosis, especially including non-wild-type strains, are necessary.

## Figures and Tables

**Table 1:** Clinical, epidemiological, and follow-up data of 37 patients with sporotrichosis treated with amphotericin B at INI/FIOCRUZ, Rio de Janeiro, Brazil, between 1998 and 2018.

Variable	Status	N = 37
**Sex**	Male	30 (81.1%)
	Female	7 (18.9%)
**Age (years)**		45.95 (15.48) ^b^
**Ethnicity**	Non-white	28 (75.7%)
	White	9 (24.3%)
**HIV**	22 (59.5%)	
**Alcoholism**	14 (37.8%)	
**Diabetes**	8 (21.6%)	
**Tuberculosis**	6 (16.2%)	
**Corticosteroid therapy**	4 (10.8%)	
**Clinical presentation**	Disseminated	35 (94.6%)
	Cutaneous disseminated	2 (5.4%)
**Affected** **organs ^a^**	Skin	36 (97.3%)
	Bones/joints	26 (70.3%)
	Upper airways	16 (43.2%)
	Central nervous system	10 (27.0%)
	Eyes	5 (13.5%)
	Lungs	3 (8.1%)
**Deoxycholate total dose (mg)**	1400.00 [50.00, 11250.00] ^c^	
**Lipid complex total dose (mg)**	5150.00 [200.00, 13900.00] ^c^	
**Total treatment time (days)**	784.00 [7.00, 3115.00] ^c^	
**Outcome**	Cure	17 (45.9%)
	Death due to sporotrichosis	9 (24.3%)
	Death unrelated to sporotrichosis	2 (5.4%)
	Loss to follow-up	3 (8.1%)
	Treatment in course	6 (16.2%)

^a^ Several patients had more than one organ affected; ^b^ mean and standard deviation; ^c^ median and range.

**Table 2:** Minimal Inhibitory Concentration of 46 *Sporothrix brasiliensis* isolates from 37 patients with severe sporotrichosis treated with amphotericin B at INI/FIOCRUZ between 1998 and 2018.

Drug	MIC ^a^ Range (µg/mL)	MIC50 ^b^ (µg/mL)	MIC90 ^c^ (µg/mL)	GM ^d^ MIC (µg/mL)
**Amphotericin B**	0.5–8	1	2	1.19
**Itraconazole**	0.5–8	1	1	0.96
**Terbinafine**	0.03–1	0.12	0.12	0.1
**Posaconazole**	0.5–8	1	2	1.19
**5-flucytosine**	≥8	8	>8	8.74

^a^ MIC—Minimal Inhibitory Concentration; ^b^ MIC50—MIC value that inhibits 50% of all the *Sporothrix brasiliensis* isolates; ^c^ MIC90—MIC value that inhibits 90% of all the *Sporothrix brasiliensis* isolates; ^d^ GM—geometrical mean.

**Table 3:** Comparison between minimal inhibitory concentration of itraconazole and amphotericin B according to clinical and epidemiological variables.

	*p* Value	
**Variable**	Itraconazole	Amphotericin B
**Sex**	0.274	0.152
**Ethnicity ^a^**	0.784	0.390
**HIV**	0.136	0.424
**Zoonotic transmission**	0.571	0.243
**Alcoholism**	**0.047**	0.153
**High blood pressure**	0.132	0.938
**Diabetes mellitus**	0.179	0.980
**Tuberculosis**	0.628	0.300
**Amphotericin B** **formulation ^b^**	0.556	0.316

^a^ White and non-white. ^b^ Deoxycholate and lipid complex. *p* value < 0.05 is highlighted in bold.

## Data Availability

The data presented in this study are available within the article.

## Supplementary Materials

The following supporting information can be downloaded at: https://www.mdpi.com/article/10.3390/jof9010049/s1, Table S1. Hazard Ratios obtained in the Cox Model for the cure event of 37 patients with sporotrichosis treated with amphotericin B at INI/FIOCRUZ, Rio de Janeiro, Brazil, between 1998 and 2018.
