# Supplementary material for: Severe Sporotrichosis Caused by Sporothrix brasiliensis: Antifungal Susceptibility and Clinical Outcomes
Source: J Fungi (Basel). 2022 Dec 28;9(1):49. doi: 10.3390/jof9010049 (PMC9864959; doi:10.3390/jof9010049)
Supplement: Supplementary file 1 [file jof-09-00049-s001.zip › jof-1992068-Table S1.pdf]

Table S1. Hazard Ratios obtained in the Cox Model for the cure event of 37 patients with sporotrichosis treated with amphotericin B at INI/FIOCRUZ, Rio de Janeiro, Brazil, between 1998 and 2018.

Variable	Category	Adjusted Hazard ratio (95% CI) *
**AMB MIC**	≥ 2	5.44 (1.48 – 20.03)
**ITR MIC**	≥ 1	4.33 (1.78 – 14.49)

*Controlled by HIV coinfection. AMB – Amphotericin B; CI – Confidence Interval; ITR – Itraconazole; MIC – minimal inhibitory concentration.
